# Feasibility of an Assessment Tool for Children’s Competence to Consent to Predictive Genetic Testing: a Pilot Study

**DOI:** 10.1007/s10897-015-9835-7

**Published:** 2015-04-26

**Authors:** Irma M. Hein, Pieter W. Troost, Robert Lindeboom, Imke Christiaans, Thomas Grisso, Johannes B. van Goudoever, Ramón J. L. Lindauer

**Affiliations:** Department of Child and Adolescent Psychiatry, Academic Medical Center, Meibergdreef 5, 1105 AZ Amsterdam, The Netherlands; Department of Clinical Methods and Public Health, Academic Medical Center, Amsterdam, The Netherlands; Department of Clinical Genetics, Academic Medical Center, Amsterdam, The Netherlands; Department of Psychology, Law and Psychiatry, University of Massachusetts Medical School, Worcester, MA USA; Department of Pediatrics, Emma Children’s Hospital, Academic Medical Center, and Department of Pediatrics, VU University Medical Center, Amsterdam, The Netherlands

**Keywords:** Sensitivity and specificity, Decision-making, Minors, Genetic testing, Informed consent, Mental competence

## Abstract

Knowledge on children’s capacities to consent to medical treatment is limited. Also, age limits for asking children’s consent vary considerably between countries. Decision-making on predictive genetic testing (PGT) is especially complicated, considering the ongoing ethical debate. In order to examine just age limits for alleged competence to consent in children, we evaluated feasibility of a standardized assessment tool, and investigated cutoff ages for children’s competence to consent to PGT. We performed a pilot study, including 17 pediatric outpatients between 6 and 18 years at risk for an autosomal dominantly inherited cardiac disease, eligible for predictive genetic testing. The reference standard for competence was established by experts trained in the relevant criteria for competent decision-making. The MacArthur Competence Assessment Tool for Treatment (MacCAT-T) served as index test. Data analysis included raw agreement between competence classifications, difference in mean ages between children judged competent and judged incompetent, and estimation of cutoff ages for judgments of competence. Twelve (71 %) children were considered competent by the reference standard, and 16 (94 %) by the MacCAT-T, with an overall agreement of 76 %. The expert judgments disagreed in most cases, while the MacCAT-T judgments agreed in 65 %. Mean age of children judged incompetent was 9.3 years and of children judged competent 12.1 years (*p* = .035). With 90 % sensitivity, children younger than 10.0 years were judged incompetent, with 90 % specificity children older than 11.8 years were judged competent. Feasibility of the MacCAT-T in children is confirmed. Initial findings on age cutoffs are indicative for children between the age of 12 and 18 to be judged competent for involvement in the informed consent process. Future research on appropriate age-limits for children’s alleged competence to consent is needed.

## Introduction

Children from families in which a causative mutation has been identified for autosomal dominantly inherited cardiac diseases may be offered predictive genetic testing (PGT) at an age when cardiologic surveillance and preventive treatment are indicated (European Society of Human Genetics 2009; Borry et al. 2009; Ross et al. 2013). In most cases the manifestations of these cardiogenetic diseases and in particular sudden death can effectively be postponed or prevented with lifestyle modifications, devices like an internal defibrillator or pacemaker, or use of medication (Smets et al. 2008). There is some preliminary evidence that such interventions might reduce risk in children (Lashley 1999). However, the penetrance of the mutations is variable and incomplete. For instance, approximately 50 % of the mutation carriers of LQTS will develop symptoms (Priori et al. 1999). For inherited cardiac diseases PGT in children is generally considered acceptable or even part of recommended care (Charron et al. 2010; European Society of Human Genetics 2009; Heart Rhythm Society (HRS) & European Heart Rhythm Association (EHRA) 2011), and it can identify individuals at increased risk of or in the early stage of a disease at a time when intervention can reduce the risk of morbidity and mortality. This article does not address the larger important ethical questions of the appropriateness of PGT in children generally. Nevertheless, the ethical debate underlines the complexity of the issue and consequently the complexity of the individual’s decision on PGT.

To illustrate the impact that cardiogenetic diseases might have, we will briefly describe the 5 syndromes dealt with in this article. Long QT syndrome (LQTS) is characterized by a prolongation of the corrected QT-interval on the electrocardiogram (ECG) and may lead to palpitations, dizziness, fainting, seizure-like fits and sudden death. Symptoms can be triggered by exertion, emotions, and loud noises in LQTS type 1 and 2, and can occur at rest in LQTS type 3. Manifestations of the symptoms can develop at all ages, but especially in LQTS type 1 and 2 occur in childhood (Smets et al. 2008). Hypertrophic cardiomyopathy (HCM) is characterized by unexplained ventricular hypertrophy and is the most common cause of sudden unexpected cardiac death in young adults, particularly in competitive athletes. Other symptoms of HCM are dyspnea, chest pain, syncope, arrhythmias, thrombo-embolic events, and heart failure (Smets et al. 2008). Brugada syndrome (BS) is a disease with typical abnormalities on the ECG and an elevated risk of sudden cardiac death. Fever as well as certain drugs can evoke the symptoms, most commonly seen in men around the age of 40 (Vatta et al. 2002). Catecholaminergic polymorphic ventricular tachycardia (CPVT) is a disease characterized by arrhythmias caused by a release of catecholamines in case of emotional upheaval, physical exercise or psychological stress (Priori et al. 2002). The first symptoms can emerge in childhood or young adulthood. Arrhythmogenic right ventricular cardiomyopathy (ARVC) is a disorder of the myocardium, that usually appears in adulthood, which increases the risk of an arrhythmia. It may not cause symptoms in its early stages, however, affected individuals may be at risk of sudden death, especially during strenuous exercise (Tichnell et al. 2014).

When clinicians do believe testing is appropriate and “in the best interest of the child” and provide testing to minors and their families, they will invite them to counsel about the disease and PGT and to give their consent to the suggested testing. This raises the question to what degree children understand the risks and benefits of PGT and the complexity of the decision, and can be deemed competent to give their consent. It is postulated that more complex decisions or decisions concerning a higher level of potential risk may require a higher level of competence, however the level of risk and complexity is not yet well quantifiable (Hein et al. 2015). Informing children and gaining their cooperation has important advantages: answering questions and helping the child to understand what to expect will help the child to make sense of the experience, prevent misunderstanding or resentment, and increase compliance (Shaw 2001). In addition, a just assessment of a child’s competence to consent is vital for striking a proper balance in order to both protect children’s interests when they are not fully able to do so themselves and to respect their autonomy when they are able to exercise it.

In clinical practice, generally the term decision-making capacity is used to describe different levels of patients’ abilities, and the term competence refers to the degree of capacity that is sufficient to allow patients to make an autonomous medical decision (Grisso et al. 1997). In medical practice, competence to consent is generally assessed implicitly and absent a standard. Clinicians tend to judge a child competent if the child’s decision conforms to their own ideas of what was in the child’s best interest (de Vries et al. 2010). The reliability of unstructured competence assessments has been poor (Appelbaum 2007) and age standards prescribed by law are the guiding principle in clinician’s competence assessments. Nevertheless, these legal age limits for deeming a child competent to consent vary widely between countries (Hein et al. 2012). In Europe different domestic laws determine whether people are competent to consent to healthcare interventions (Stultiens et al. 2007). Some countries consider autonomous decision-making lawful from the age of 18 onwards, and in other countries people are allowed to take healthcare decisions from a fixed age below legal majority, for instance, 12 years in the Netherlands and 15 years in Denmark (Stultiens et al. 2007). Most Canadian provinces and Switzerland apply a flexible system, stating that anyone who is capable can give informed consent, whereby competence is evaluated on a case-by-case basis (Stultiens et al. 2007). In the United States, generally speaking, it often falls to parents or legal guardians to provide informed permission for medical decisions, and children under the age of 18 are to give assent (American Academy of Pediatrics Committee on Bioethics 1995). Assent includes that physicians give serious consideration to the developing capacities of older children and adolescents for participating in decision-making, and solicit their affirmative agreement (American Academy of Pediatrics Committee on Bioethics 1995). In our study, in order to deal with discrepancies between the local law and international jurisdictions, we studied children’s capacities for deciding on consent regardless of their age.

The capacities of children to participate in medical decision making remains inconclusive and there is not a well-established assessment approach, neither have the legally set age limits been systematically investigated. Generally, the accepted standard in adults for assessing capacity to make treatment decisions consists of an unstructured judgment by an expert, trained in the four criteria that reflect the standards to be weighed in most jurisdictions: understanding, reasoning, appreciation, and expressing a choice (Hein et al. 2014a).

Empirical studies on children’s capacities to make treatment decisions are very limited. As far as we know, only 3 studies have been conducted using a structured instrument that addresses all four relevant criteria, which in all cases was the MacArthur Competence Assessment Tool for Treatment (MacCAT-T) (Grisso et al. 1997). Chenneville investigated the MacCAT-T in a sample of youth with an average age of 17 years, with HIV (Chenneville et al. 2014). A limitation of this study is that previously established cutoff scores were used (Aydin and Sehiralti 2014) which were established in a population of adult psychiatric patients and not evaluated in a sample of minors. Turrell and colleagues used the MacCAT-T in a comparative study on capacities to consent in adolescents with anorexia nervosa and healthy controls and found group differences: adolescents with anorexia nervosa tended to experience more problems in reasoning about treatment than healthy controls (Turrell et al. 2011). Schachter and colleagues assessed understanding by means of a modified version of the understanding section of MacCAT-T. Results suggested that the majority of adolescents with ADHD have an understanding similar to that of their parents (Schachter et al. 2011). None of these studies tested the reliability and validity of the structured assessment instrument against a reference standard. Empirical research on children’s competence to consent is still a novel area.

Therefore, the aim of our pilot study is evaluating feasibility of a standardized competence assessment tool for children in PGT by modifying the MacCAT-T (Grisso et al. 1997), and initially investigating accuracy against a reference standard and cutoff ages for competence to consent.

## Methods

### Participants

Participants were pediatric outpatients between 6 and 18 years of age visiting the clinical genetics department at the Academic Medical Center in Amsterdam, the Netherlands, who were prospectively enrolled. They were referred by physicians for being at risk for an autosomal dominantly inherited cardiac disease. Exclusion criterion was not speaking Dutch. The study protocol was approved by the institutional review board and written informed consent was obtained from all individual participants included in the study, or a parent or legal guardian.

### Instrumentation

As the index test for assessment of abilities related to competent consent, we used the MacCAT-T, developed by Grisso and Appelbaum in 1998 (Grisso et al. 1997). PGT in fact concerns diagnostic testing, however it takes place in a treatment context and therefore the MacCAT-T is the most appropriate instrument. The MacCAT-T measures the four aspects of decision-making capacities by operationalizing the four criteria into a semi-structured interview format: (1) understanding the disclosed information about the nature of the disease and the proposed intervention; (2) reasoning in the process of deciding about the proposed intervention, with a focus on abilities to compare alternatives in the light of their consequences; (3) appreciation of the effects of the intervention (or failure to undergo the intervention) on patient’s own situation; and (4) expressing a choice about the intervention. Information disclosure required for informed consent is combined with an assessment of the patient’s capacities. In this study the information disclosure was adapted to the specific cardiogenetic disease that a participant was tested for. The method provides scores for each subscale: 0–6 for understanding, 0–4 for appreciation, 0–6 for reasoning, and 0–2 for expressing a choice. The method does not offer a total score or a cutoff for competence, but the scores on the subscales need to be weighed by the interviewer. The MacCAT-T takes approximately 15 min administration time, and some additional minutes for scoring. It receives empirical support in adult populations of mentally compromised patients (Hein et al. 2014a). The MacCAT-T was translated in Dutch, and translated back in English, by a professional translator. The version used was approved by the original author (T.G.). The Dutch version was modified for children which included the use of simple language to be understood by children of elementary school age. The interview was read out aloud to participants to exclude interference of children’s reading levels. Furthermore, in the child version of the MacCAT-T we added questions on the influence of social relationships (Hein et al. 2012). In the reasoning domain, “How do you think your parents will feel about you deciding to have this diagnostic test or deciding not to have it? And how do you think your friends will feel about it?” has been added (proprietary issues preclude publication of the version used).

Although a gold standard for competence does not exist, we examined whether using a structured assessment instrument instead of an expert judgment would be possible without compromising accuracy. Usually, agreement is poor between unstructured clinical competence judgments, and often no better than chance (Hein et al. 2014a). Providing clinicians with information regarding the legal standards improves their judgments and significantly increases the inter-rater agreement (Hein et al. 2014a). These legal standards embody the four capacities: to communicate a choice, to understand the relevant information, to appreciate the medical consequences of the situation, and to reason about treatment choices. Clinicians aware of these relevant criteria are generally considered to establish the reference standard (Hein et al. 2014a). However, limitations of this approach may include discordance of expert competence judgments, leading to inconsistencies in the reference standard. Thus, poor performance of the MacCAT-T could either result from imperfections in the reference standard, or from an inaccurate assessment of competence based on the examiner’s use of the MacCAT-T.

### Procedures

Children and parents were informed by a genetic counselor or clinical geneticist on PGT (Christiaans et al. 2008), according to the consensus documents on genetic testing in children of the Dutch and European Societies of Human Genetics. The counselling session was preceded by a telephone call from the psychosocial worker with the parents to already discuss how the children had reacted on the information provided by the parents about the genetic disease, if they had experienced symptomatic disease in close relatives and if there were any psychosocial issues to be aware of in the counselling session. The counselling session at the outpatient clinic comprised issues necessary for informed decision-making, including the characteristics of the disorder tested for, the possible treatment options, the possible drawbacks of testing, and possible psychosocial consequences. Parent(s) and children were asked if they consented to PGT. This conversation was videotaped and served as the basis for establishing the reference standard (see below). Usually at the same day, at most within 2 weeks, an interviewer from a panel of experts (listed below) administered a MacCAT-T interview to the child. This interview was also videotaped, and rated afterwards.

The panel of 7 experts (including I.M.H., P.W.T., I.C., and R.J.L.L.) consisted of a clinical geneticist, child psychiatrists, child psychologists, and a social worker. The experts were trained in judging competence to consent by the 4 relevant criteria, and jointly practiced through rating 3 videos of the conventional informed consent conversation. In addition, the experts were instructed on rating the MacCAT-T, and practiced together by rating 2 videos of MacCAT-T interviews. Next, each member of the panel independently rated a number of conventional informed consent conversation videos and a number of MacCAT-T interview videos that were presented in random order and reciprocally blinded. Each MacCAT-T interview video was rated by 3 different experts. For all videos, the experts gave their judgment consisting of 1 of the following 4 categories: very likely competent, probably competent, probably incompetent, and very likely incompetent. We considered competence to be present when an expert gave a judgment of very likely or probably competent. The experts were not informed about the age of the children. For establishing the reference standard, each video from the conventional informed consent conversation was rated by 2 different experts, and also the clinical geneticist gave his/her judgment of the child’s competence, adding up to 3 judgments.

The cognitive level of the children was assessed by the Wechsler Nonverbal Scale of Ability short version (WNV). The WNV is a clinical instrument for examining cognitive capacities of children and adolescents aged 4 to 21, which is suitable for the general population as well as for children with cultural, linguistic, educational or socio-economic varying backgrounds. The subtests do not invoke verbal capacities as instructions are made by pictograms, and the validity and reliability of the short version are good. The WNV was administered by trained certified professionals (special education or psychology graduates) under supervision of a senior professional.

### Data analysis

Competence was considered present when at least 2 out of 3 judgments were positive, for both the expert judgments establishing the reference standard, and the ratings based on the MacCAT-T. Agreement between the reference standard and the MacCAT-T based competence classification (accuracy of the MacCAT-T-based competence classifications) was expressed as the raw percentage agreement.

Reproducibility of the MacCAT-T total and subscale sum scores as obtained by 3 ratings on the MacCAT-T, was estimated using intraclass correlation coefficients (ICC, model 1, single measure).

Agreement between the 3 ratings of the experts, and between the 3 ratings based on the MacCAT-T, was expressed as the raw percentage.

Independent samples *t*-test was used to test the difference in mean ages between competent and incompetent children on the reference standard. Best discriminating cutoff ages for competence on the reference standard were estimated using receiver operator characteristic curve (ROC) analysis, with area under the curve (AUC) exceeding .70 considered adequate for the estimation of age cutoff.

## Results

Between January 1, 2013 and January 1, 2014, 23 children were eligible. Of them, 6 did not participate for different reasons, concerning time constraints in 2 cases, elevated stress in 2 cases, and no clear reason in 2 cases. Non-participants were 4 males, mean age 10 years, and 2 females, mean age 12 years. The characteristics of the 17 included children are listed in Table 1. The age range of the participants was between 6 and 17, mean 10.9, variance 6.7. The Intelligence Quotient as measured by the WNV ranged from 89 to 126, with a mean of 107.5.

**Table 1:** Baseline characteristics and outcomes of competence classifications on reference standard and MacCAT-T

Child no.	Disease tested for^a^	Age in years (male/female)	Expert classification (competent: incompetent)	MacCAT-T score, (subscale scores U/A/R/C)^b^	MacCAT-T classification (competent: incompetent)
1	HCM	6 F	I (0:3)	29 (21/2/3/2)	C (2:1)
2	LQTS	7 M	I (0:3)	30 (21/4/4/2)	I (1:2)
3	BS	9 M	I (0:3)	36 (23/4/7/2)	C (3:0)
4	HCM	9 M	C (3:0)	32 (18/4/8/2)	C (3:0)
5	HCM	10 M	C (2:1)	31 (21/4/4/2)	C (2:1)
6	HCM	10 M	C (2:1)	35 (22/4/7/2)	C (3:0)
7	HCM	10 F	C (3:0)	38 (25/3/8/2)	C (3:0)
8	HCM	10 F	C (2:1)	36 (23/2/9/2)	C (3:0)
9	HCM	11 M	C (2:1)	36 (22/4/8/2)	C (3:0)
10	HCM	11 F	I (1:2)	29 (18/2/7/2)	C (2:1)
11	HCM	11 M	I (1:2)	38 (25/4/7/2)	C (3:0)
12	HCM	12 M	C (2:1)	30 (20/4/4/2)	C (2:1)
13	CPVT	12 M	C (2:1)	38 (24/4/8/2)	C (3:0)
14	CPVT	13 F	C (2:1)	34 (24/3/5/2)	C (2:1)
15	LQTS	13 F	C (3:0)	40 (25/4/8/2)	C (3:0)
16	ARVC	14 F	C (3:0)	40 (26/4/8/2)	C (3:0)
17	ARVC	17 M	C (3:0)	38 (25/4/7/2)	C (3:0)
					76 % agreement MacCAT-T vs expert

^a^ *ARCV* arrhythmogenic right ventricular cardiomyopathy, *BS* Brugada syndrome, *CPVT* catecholaminergic polymorphic ventricular tachycardia, *HCM* hypertrophic cardiomyopathy, *LQTS* long QT syndrome

^b^Mean scores from three raters, *U* understanding, *A* appreciation, *R* reasoning, *C* choice

By the reference standard 12 (71 %) children were classified competent, by MacCAT-T classification 16 (94 %) children. In 24 % of the children, the expert raters classified the child as incompetent and the examiners using the MACAT-T did not. The other way around did not occur. Overall agreement was 76 %.

MacCAT-T total scores inter-rater agreement coefficient was .95. Inter-rater agreement on subscale scores were .93 for understanding, .91 for appreciation, .91 for reasoning and total agreement for choice.

Agreement between all 3 ratings on the reference standard occurred in 8 cases (47 %), and on the MacCAT-T based classification the 3 ratings showed agreement in 11 cases (65 %).

On the reference standard, mean age of children judged incompetent was 9.3 years and for those judged competent 12.1 years (*p* = .035).

Age as a predictor of competence on the reference standard showed AUC .80 (95 %; .55–1.00). Cutoff age for being judged competent with 90 % sensitivity was 10.0 years and with 90 % specificity 11.8 years.

## Discussion

Results of the current study confirm feasibility and show initial indications for reliability and validity of the MacCAT-T in children eligible for PGT: inter-rater agreement on scores was high, and agreement between MacCAT-T based competence classifications and the reference standard was adequate, although not decisive. By using the MacCAT-T, children were more often classified as competent than by the reference standard.

Age cutoffs for presumed competence to consent to PGT in this sample, based on the reference standard, were: children of 11.8 years and above were very likely to be considered competent to consent to PGT, and children of 10.0 years and younger were most probably not competent to consent. Earlier work showed that the modified MacArthur Competence Assessment Tool for Clinical Research was valid and reliable for use in children (Hein et al. 2014b) and in a population of 161 pediatric patients eligible for research participation, children older than 11.2 years were generally found competent to consent and children younger than 9.2 years incompetent (Hein et al. 2014b). Obviously, children’s competence to consent to treatment and their competence to consent to clinical research are not the same. It has been stated that consent to participation in research must be a more stringent process than consent to treatment, because the research participants are generally not asked to participate for their individual benefit, but to help improve general health care (Lind et al. 2003). Additionally, the level of complexity and risk of the decision on PGT, which has not been quantified in this study, may affect the level of competence required and thereby the resulting age-limit in this specific population. Nevertheless, the fact that the present findings are consistent with previously found age cutoffs in children regarding their competence to consent to clinical research, increases support for the results.

The rate of disagreement between the competence ratings was high, both for expert judgments and for the MacCAT-T based judgments. Even so, where the expert judgments disagreed in most cases, the use of the MacCAT-T led to an increased agreement. Factors that explain the high rate of disagreement between judgments, no matter what assessment method was used, might be related to normative aspects and difficulties in assessing developmental aspects in children. Although decision-making competence may be a matter of minor differences (Buchanan and Brock 1990), the competence judgments require a definitive assessment of whether competence is present or not. It is still under debate whether a threshold for competence can be established based on the sum of the different decision-making capacities (Vellinga 2006). Especially in children, development of different domains relevant for competent decision-making may not occur simultaneously, which may complicate the assessment of a child’s competence.

### Practice Implications

For the clinical practice of PGT, no definitive conclusions can be drawn from this study’s results. Yet, the results indicate preliminarily that clinical judgments of competence to consent to treatment can be present in children under the age of 18, even when it concerns a complex decision regarding PGT. Moreover, in our small sample, all children of 12 years and above were considered competent to consent to PGT, independent of the assessment method used. Taking into account that understanding of the relevant medical information is critical for competent decision-making, it deserves attention to supply even young children with adequate information tailored to their developmental stage and comprehension level (Hein et al. 2012) in order to optimally involve them in the informed consent process.

### Study Limitations

A salient limitation of this study concerns the small sample size and wide age range, which complicate an exhaustive analysis of the data. Furthermore, poor reliability among experts forming the reference standard is a significant limitation, as it was the benchmark that the MacCAT-T results were compared to. The fact that all but 1 child in the sample was rated as competent by using the MacCAT-T should be noted, as this could relate to limited utility when using the MacCAT-T. Although participants of a wide age range were recruited, the obtained sample contains for the greater part children between 9 and 14 years of age, thus the generalizability of the results beyond this age group must be considered with caution.

### Research Recommendations

More empirical research on children’s capacities to consent to treatment is needed, in order to provide objective data to underpin a just age limit for alleged competence. In addition, an accurate assessment instrument is needed to substantiate competence judgment in individual cases. Exploration of the psychometric properties of the MacCAT-T in a larger sample can enable estimation of a cutoff score above which competence is more likely. More extended research should be directed especially at pediatric populations where competence issues can become problematic. Examples of such situations comprise children older than the legal age for competence who refuse a recommended medical treatment, for instance children with anorexia nervosa who refuse tube feeding, or children with renal insufficiency who refuse dialysis. Also children younger than the legal age for competence who wish for a certain treatment, like children eligible for medical interventions for gender dysphoria, must be considered. Future research should address the issue of the presumed higher requirements for competence when decisions are more complex or carry more potential risk.

## Conclusion

Our present results confirm that the MacCAT-T is promising for standardizing competence assessment in children in treatment situations. The strength for using the MacCAT-T includes high interrater agreement, and the consistency in MacCAT-T results compared to the expert judgments lends additional support to the use of the instrument. The reliability and validity of the MacCAT-T must be demonstrated in a larger sample of children.
